# Potential Involvement of Fructosylated Human Insulin and Serological Evidence in Subclinical Autoimmune Activity in Type 2 Diabetes Mellitus

**DOI:** 10.2174/0115733998451601260128044731

**Published:** 2026-03-12

**Authors:** Ali Raza, Safia Habib, Saba Noor, Rizwan Ahmad, Mortha Chandni, Moinuddin, Riaz Mahmood, P.F. Rahaman, Arif Ahmad

**Affiliations:** 1 Department of Biochemistry, Jawaharlal Nehru Medical, College Faculty of Medicine, Aligarh Muslim University, Aligarh, 202002, Uttar Pradesh, India;; 2 Center for Interdisciplinary Research in Basic Sciences, Jamia Millia Islamia, Jamia Nagar, New Delhi-110025, India;; 3 Department of Biochemistry, Faculty of Life Sciences, Aligarh Muslim University, Aligarh, 202002, Uttar Pradesh, India;; 4 Zoology Section, School of Sciences, Maulana Azad National Urdu University, Hyderabad, 500032, Telangana, India

**Keywords:** Diabetes, fructose, insulin, aggregation, neoepitopes, autoimmunity, ELISA

## Abstract

**Introduction:**

Type 2 Diabetes Mellitus (T2DM) involves chronic inflammation and recurrent hyperglycemia, often linked to excessive fructose intake. Some patients develop autoantibodies, indicating immune dysfunction. Fructose promotes protein glycation and the formation of Advanced Glycation End-products (AGEs), which may expose novel epitopes recognized as foreign. This study examined the antigenicity of fructosylated human insulin and its impact on lymphocytes in diabetic patients.

**Methods:**

Fructosylated insulin was characterized using spectroscopic techniques, with aggregation assessed by ThT, ANS, and DLS, and conformational changes analyzed *via* CD spectroscopy. Biochemical and biophysical methods confirmed the presence of structural alterations. Antigenicity was evaluated using ELISAs in sera from 40 healthy controls and 40 newly diagnosed T2DM patients, with ROC and AUC analyses determining prognostic relevance.

**Results:**

Concentration-dependent increase in AGE-specific fluorescence, increased hydrophobicity, and significant loss of lysine (48.6%) and arginine (58.8%) residues, accompanied by conformational changes. Direct binding ELISA revealed statistically significant reactivity (*p* ≤ 0.05) with diabetic sera. Competition ELISA demonstrated 55% ± 3.7% inhibition for fructosylated insulin compared to 31.4 ± 5.1% for native insulin. IgGs isolated from diabetic sera exhibited 96.67% specificity for fructosylated insulin, with sensitivity and specificity analyses yielding AUC = 1.000 (*p* < 0.01).

**Discussion:**

Fructosylation causes changes in insulin chemistry, which leads to altered structural, conformational, and antigenic potential in T2DM patients.

**Conclusion:**

Screening for anti-fructosylated insulin antibodies may help identify T2DM patients with hyperglycemia-associated, subclinical autoimmune activity linked to abnormal insulin responses.

## 1 INTRODUCTION

Type 2 Diabetes Mellitus (T2DM) is a global health epidemic affecting 537 million individuals aged 20 to 79. It is projected to impact 643 million by 2045 [1]. Approximately 247 million diabetes cases remain undiagnosed worldwide, with most originating from low- to middle-income regions like South Asia. According to the International Diabetes Federation (IDF), India ranks second among countries with the highest number of diabetic cases, currently at 77 million, a figure expected to rise to 134 million by 2045 [2]. Over the past decade, the increasing global prevalence of Young-Onset Diabetes (YOD)-diagnosed before age 40-has emerged as a significant health concern [3]. YOD has been linked to rising obesity rates and disruptions in β-pancreatic cells [4]. In South Asian countries like India, diabetes onset is believed to occur 2 decades earlier than in Western nations. Such cases typically feature a lower Body Mass Index (BMI), a higher predisposition to fat, widespread inflammation, and insulin resistance [5]. Young individuals with diabetes are at greater risk of developing metabolic disorders, which may lead to vascular complications and ultimately cardiovascular events, in contrast to those diagnosed later in life [6]. As previously discussed, countries with low to middle socioeconomic status represent a disproportionately high number of diabetes cases globally. This uneven distribution of diabetes is often attributed to dietary shifts linked to a region's socioeconomic status [7]. Among the risk factors associated with diabetes, dietary habits play a crucial role. Several nutritional factors can disrupt metabolic balance and increase blood glucose levels. Prolonged elevated blood glucose levels, or hyperglycemia, can glycate proteins, a critical component of metabolic disruption. Since dietary shifts are commonly regarded as a major factor in the gradual transition from a non-diabetic to a prediabetic population, the inclusion of affordable and appealing sweet food items, such as High-Fructose Corn Syrup (HFCS), has contributed to the increasing prevalence of diabetes. HFCS comprises 55% fructose, 42% glucose, and 3% additional sugars, making it the most utilized alternative to sucrose. Consumption of soft drinks and beverages containing HFCS is known to trigger weight gain, hypertrophy of adipose cells, elevated fasting blood sugar, and increased circulating triacylglyceride (TAG) levels [8-10]. The adoption of a processed diet inevitably leads to higher consumption of processed sweetened foods and beverages, which contain excessive amounts of fructose from HFCS. Once ingested, fructose is transported into cells *via* two key glucose transporters, GLUT5 and GLUT2, which are members of the SLC2A family of proteins. GLUT5 primarily transports fructose across the apical membrane, while GLUT2 facilitates transport from the cytosol into the bloodstream [11]. Furthermore, fructose is also synthesized endogenously through the activation of the polyol/sorbitol pathway as a mechanism to cope with high glucose levels in the body. In this pathway, glucose is first converted to sorbitol by aldose reductase using NADH, followed by its conversion to fructose by sorbitol dehydrogenase [12]. Activation of this pathway under hyperglycemic conditions leads to excessive NADH utilization, sorbitol accumulation, overproduction of NADH, and endogenous fructose generation, all associated with diabetes progression [13]. Excessive fructose intake, along with its endogenous synthesis, raises the risk of abnormal protein glycation, which can result in the accumulation of Advanced Glycation End-products (AGEs). Fructose-mediated glycation, also termed fructosylation, involves a series of non-enzymatic reactions between fructose and the amino groups of target proteins, leading to the formation of highly toxic AGEs such as N-carboxymethyllysine, carboxyethyl-lysine, and pentosidine. Glycation/fructosylation causes a loss of secondary protein structure and promotes oligomerization [14]. Since insulin is vital for glucose homeostasis, its structural compromise during hyperglycemic conditions exacerbates metabolic derangements.

Thus, understanding its role in fostering pro-diabetic conditions is essential. Our previous research demonstrated that fructose interacts with insulin under physiological conditions. Molecular docking, molecular dynamics simulations, and *in vitro* studies have indicated that fructose not only forms a complex with insulin but also reacts irreversibly, producing fructosamine and AGEs. Following fructosylation, these significant chemical disturbances lead to compromised insulin helicity, aggregation, and the formation of amyloid-like structures in purified insulin [15, 16]. There is evidence that protein aggregations can result in enhanced immunogenicity [17-20]. Moreover, as T2DM is typically characterized by subclinical inflammation [21], understanding the inflammatory status and the role of fructosylated insulin in antibody response, as well as its effect on isolated peripheral lymphocytes, may help identify it as one of the primary pathogenetic factors driving T2DM progression, correlated with heightened levels of chronic proinflammatory cellular status. Immune cells serve as prominent sources of cytokine production, and under hyperglycemic conditions, the system is compelled to produce more fructose, increasing the probability of fructosylating proteins (here, insulin). Therefore, the effect of fructosylated insulin on lymphocytes will further promote chronic inflammation. This work primarily focuses on identifying the antigenicity of fructosylated human insulin in diabetic patients and, secondarily, on determining the effect of fructosylated insulin on lymphocytes that contributes to the altered inflammatory status of diabetic individuals, potentially leading to the rapid progression of associated chronic complications.

## 2 MATERIALS

Human insulin (91077C), bovine serum albumin (BSA), Thioflavin-T, 8-Anilinonaphthalene-1-sulfonic acid (ANS), 2, 4, 6-trinitrobenzene sulphonic acid (TNBS), 2,4-dinitrophenylhydrazine (DNPH), anti-human IgG alkaline phosphatase conjugate, Tween-20, para-nitrophenyl phosphate (PNPP), 2,7-dichlorofluorescein diacetate (DCFH-DA), propidium iodide, and dialysis tubing were purchased from Sigma-Aldrich, St. Louis (Missouri, U.S.A.). Flat-bottom Maxisorp ELISA modules were purchased from NUNC, Denmark. All other chemicals were of analytical grade.

## 3 METHODOLOGY

### 3.1 Fructosylation of Human Insulin

Increasing fructose concentrations (5, 10, 25, and 50 mM) were used to treat human insulin (56 µM) in 50 mM sodium phosphate buffer (pH 7.4) under sterile conditions at 37 ºC for 10 days. After the incubation period, extensive dialysis was performed against 3L of 10 mM sodium phosphate buffer (pH 7.4) at 4°C. Dialysis tubes (D7884) were purchased from Sigma-Aldrich, USA, which separate molecules with a molecular weight ≤1,200 from the compounds having a molecular weight >2,000. A high buffer-to-sample ratio (> 300-fold) was maintained to preserve the concentration gradient. The untreated insulin and treated insulin samples were dialysed for 1-2 h before the dialysis buffer was changed at room temperature. This step was repeated once again before the final dialysis, which was done at 4°C overnight. Insulin samples not treated with fructose served as a control for comparative analysis and are termed native insulin.

Based on previously published studies [15-17], the antigenicity of fructosylated insulin (25 mM fructose) was checked against T2DM sera. Insulin fructosylated with 5- and 25 mM fructose was used to assess its effect on isolated human lymphocytes.

### 3.2 Characterization of the Epitope Changes on Fructosylated Human Insulin

#### 3.2.1 UV-Vis Absorbance and Fluorescence Spectroscopy

UV-Vis spectroscopy was performed on the fructosylated insulin along with an appropriate control. A wavelength range of 200 to 400 nm was selected to scan the samples. The following equation was used to calculate the percentage gain in hyperchromicity [22]. Spectral absorption data were recorded at 280 nm, and the percent change in chromicity was calculated using Eq. (**1**).


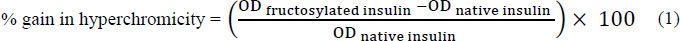


Intrinsic fluorescence quenching of fructosylated insulin was studied using a Shimadzu RF-5301-PC spectrofluorometer (at 25±0.1°C). Excitation of the samples was performed at 275 nm, and emission was recorded over 290-400 nm [21]. The percent loss in fluorescence intensity (FI) for insulin samples treated with fructose was calculated using Eq. (**2**).


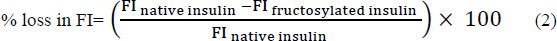


#### 3.2.2 ThT and ANS Binding Studies to Identify Aggregation and Extent of Hydrophobicity of Fructosylated Human Insulin

To detect the presence of fibril-like structures in all samples, Nilsson’s procedure was adopted [23]. Thioflavin-T (ThT) is a well-known fluorescent chemical that is used to target protein aggregates, especially amyloid-like fibrils, which, when exposed to ThT, show strong binding and fluorescence [24]. ThT is a dye that, when excited at 350 nm, gives a maximum emission of 450 nm. To 20 μM of ThT, 5 μl of each sample was incubated in a molar ratio of 1:2 in 50 mM PBS (pH 7.4) for 1 hour at 25°C. Fluorescence intensities were then measured using a spectrofluorometer, using a cuvette with a 1 cm path length and a slit width of 5 nm. Native insulin + ThT was used as a blank.

Changes in fluorescence were recorded, and the differences in fluorescence intensities (FI) between fructosylated insulin samples and native insulin samples were expressed as a percentage using Eq. (**3**).


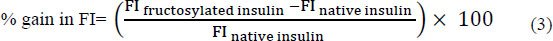


To understand the behaviour of hydrophobic sites of the insulin molecule, 8-Anilinonaphthalene-1-sulfonic acid (ANS) was used. After preparing an ANS stock solution of 20 mM in ethanol, its concentration was determined by spectrophotometry using a molar extinction coefficient of 5000 M^-1^ cm^-1^ at 350 nm [25]. Insulin samples and ANS were mixed in a molar ratio of 1:50, and the samples were excited at 380 nm. The emission was recorded between 400 and 600 nm. Native insulin + ANS was used as a blank.

The difference in ANS-binding intensity between the fructosylated insulin sample and native insulin was calculated in terms of percentage using the same formula. The experiments were carried out in duplicates, and the results are reported from the mean of the experimental values.

#### 3.2.3 Estimation of Free Lysine and Arginine Residues

The free ε-amino groups in native and fructosylated insulin were estimated using 2, 4, 6-trinitrobenzene sulphonic acid (TNBS). Samples were diluted in 100 mM sodium bicarbonate buffer (pH 8.5). This was followed by adding 0.25 ml of 0.01% (v/v) TNBS, and then incubating at 37 ºC for 2 hours. Finally, the samples were solubilized using 0.25 ml SDS (10%). The last step was the addition of 0.1 ml of 1N HCl before recording the absorbance at 340 nm.

Similarly, free arginine residues were estimated fluorometrically using 9,10-phenanthrenequinone. To each 50 µl reaction sample, 75 µl of 2N NaOH and 450 µl of 150 µM 9,10-phenanthrenequinone were mixed and incubated for 1 hour at 30 ºC. This was followed by the addition of 75 µl of 1.2 N HCl. After excitation at 312 nm, the fluorescence emission was observed at 395 nm [26].

#### 3.2.4 Estimation of Carbonyl Content

Reactive carbonyl groups were determined by using 2,4- dinitrophenylhydrazine (DNPH) in native and fructosylated insulin samples [22]. After recording maximum absorbance at 360 nm, the total carbonyl content of insulin incubated with 5, 10, 25, and 50 mM fructose concentration was measured. Using the molar absorption coefficient of 22,000 M^-1^cm^-1^. The concentration is reported in nmol/mg insulin.

#### 3.2.5 Dynamic Light Scattering (DLS)

To study the aggregate formation, insulin was incubated with 5, 10, 25, and 50 mM fructose. Samples were analyzed by dynamic light scattering on a Zetasizer device (Nano ZS; Malvern Instruments, UK) using a 633 nm wavelength He-Ne laser operating at a fixed angle of 173º. Throughout 8 runs, each spectrum consisted of an average of 3 observations. Malvern Zetasizer software (Malvern Instruments) was used for the examination and analysis of the machine data [27].

#### 3.2.6 Circular Dichroism (CD) Spectroscopy

To analyse changes in insulin's secondary structure due to fructosylation, CD spectroscopy was performed. The experiment was conducted using a CD spectropolarimeter (J-1500, JASCO, Japan) using D-10 camphor sulphonic acid for calibration. Spectral range of 195-250 nm was set for further analysis. At 208 and 222 nm, molar residue ellipticities (MRE) were recorded at 208 and 222 nm to identify secondary structural changes using the equation below:


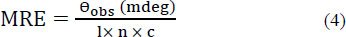


Here, Ɵ_obs_ represents the CD units in mdeg, n denotes the number of amino acids in the protein, l stands for the path length in cm, and c indicates the concentration of protein in moles/l. Native insulin served as a control for the above analysis.

### 3.3 To Screen for the Presence of Anti-Fructosylated Human Insulin Antibody in the Peripheral Blood by Direct Binding ELISA

#### 3.3.1 Collection of Venous Blood Samples

After obtaining informed and written consent, venous blood was collected from age-matched (20-40 years) 40 healthy individuals (control) and 40 newly diagnosed T2DM patients, before any treatment, from the OPD of Rajiv Gandhi Centre for Diabetes and Endocrinology, Jawaharlal Nehru Medical College, Aligarh Muslim University, Aligarh. The patients were classified as diabetic according to the American Diabetes Association (ADA, 2023). Patients with T1DM, on oral hypoglycemics, insulin therapy, hormone replacement therapy, alcoholics, smokers, and females with gestational diabetes were excluded from the study. All the study participants were made to sign an informed consent form. The work was duly approved by the Institutional Ethical Committee (IEC), Jawaharlal Nehru Medical College and Hospital, Faculty of Medicine, Aligarh Muslim University, India. Under the National Ethics Committee Registry for Biomedical and Health Research, NECRBHR DHR ICMR and Central Drugs Standard Control Organization (CDSCO), Ministry of Health and Family Welfare, Govt. of India. (Ref. No.: IECJNMC/1036). The IEC committee has reviewed and approved all experiments conducted on human blood samples. The study has followed all relevant guidelines/regulations governing human subjects and was conducted in accordance with the Declaration of Helsinki. Among the participants of the control group, 19 were females (47.5%), and 21 were males (52.5%). The T2DM case group consisted of 18 females (45.0%) and 22 males (55.0%). The control group had glycated haemoglobin (HbA1c) of 5.5100 ± 0.71044. In contrast, the case group had a mean HbA1c of 6.8650 ± 0.57179. Blood was collected in AcCuvet-PLUS/clot activator tubes and incubated for 30 min at 37ºC to allow clotting. To separate the sera, all tubes were centrifuged at 3,000 rpm. The serum samples were decomplemented by heating at 56 ºC for 30 min and stored at -20 ºC with 0.2% sodium azide for further analysis. Both cases and controls were screened for the presence of anti-fructosylated-insulin autoantibodies through direct binding ELISA using 1:100 serum dilutions.

#### 3.3.2 Direct Binding Enzyme-Linked Immunosorbent Assay (ELISA)

A standardized, established protocol from our lab was used for direct binding ELISA [27]. Sera was isolated from the study subjects and diluted to 1:100. Polystyrene Maxisorp microtiter wells were coated with 10 µg/ml of antigens (native insulin and insulin fructosylated with 25 mM fructose), and kept for two hours at 37°C, followed by overnight incubation at 4°C. Extensive washing was performed with TBS-T (20 mM Tris, 2.68 mM KCl, and 150 mM NaCl, containing 0.05% Tween-20), pH 7.4. Diluted sera (100 μl) were coated in the respective wells and incubated at 37°C, followed by extensive washing. Nonspecific and unoccupied sites in the wells were blocked with 200 μl of 1.5% Bovine Serum Albumin (BSA) dissolved in TBS (10 mM Tris, 150 mM NaCl), pH 7.4, and kept at 37°C for 6 hours, then overnight at 4°C. Bound antibodies were analyzed using an anti-human-IgG alkaline phosphatase conjugate, and 500 μg p-nitrophenyl phosphate dissolved in 1 ml of carbonate-bicarbonate buffer (pH 8.6) was coated as substrate. Absorbance was recorded at 410 nm using an iMark-Bio-Rad microplate reader (United States of America). The results were calculated as the mean difference in absorbance values between the test and control wells [*Abs_(test)_* - *Abs_(control_*_)_]_._ Each set was run in duplicates, and the results were represented as mean ± standard deviation.

#### 3.3.3 Isolation of IgG from the Sera

IgGs were isolated using a Protein A-Agarose affinity column according to the protocol described by Abidi *et al.* (2020) [28]. Absorbance of IgGs in fractions of 3 ml was recorded at 280 nm. The eluted IgGs were quantified by considering 1.4 OD equivalent to 1 mg/ml, and the purity check of the eluted IgGs was performed on a 10% SDS-polyacrylamide gel under both reducing and non-reducing conditions. The IgGs were dialyzed using PBS (pH 7.4). Sodium azide (0.1%) was added as a preservative to prevent bacterial contamination. Aliquots were stored at -20ºC.

#### 3.3.4 Inhibition ELISA to Check the Binding Specificity of Isolated IgG

To study the binding specificity of the IgGs isolated from healthy and diabetic serum samples, each was subjected to inhibition ELISAs using the method reported by Talha *et al.* (2021) [22]. The plates were coated with 100 μl of the antigens listed above at 37°C for 2 hours, followed by overnight incubation at 4°C. Native and fructosylated insulin were used as inhibitors with concentrations ranging from 0 to 20 μg/ml. At each concentration, the inhibitor was mixed with 100 μl of 40 μg/ml of anti-fructosylated IgG. The mixtures were incubated at 37°C for 2 hours and overnight at 4°C. Subsequently, the immune complexes were used to coat the wells instead of serum. The remaining steps were the same as mentioned for the direct binding ELISA. The calculation of percent inhibition is done using Eq. (**5**).


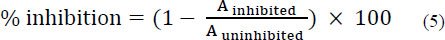


#### 3.3.5 Cytokines Estimation Involved in the Subclinical Autoimmune Responses

Serum levels of IL-6, IL-10, IL-17, and IL-18 were measured using enzyme-linked immunosorbent assay (ELISA). The GENLISA Human ELISA Kits were purchased from Krishgen BioSystem (REF KB1145). The absorbance at 450 nm was recorded with an automated ELISA plate reader (Bio-Rad, Japan; Model No. iMark 10630). Each sample was tested in duplicate, and the final absorbance was reported as the mean ± standard deviation of the duplicate readings, according to the manufacturer's protocol.

#### 3.3.6 Statistical Analysis

Statistical analysis was performed using SPSS software, Windows 20.0 (SPSS Inc., Chicago, IL), and GraphPad Prism 8.0 (GraphPad Software Inc., San Diego, CA). Data are presented as means ± standard deviations or medians (interquartile ranges) based on the data and variable types. A Student's t-test, or the Kolmogorov-Smirnov test, was performed to compare the two groups and assess distributional differences and significance. P values are reported and considered significant at ≤ 0.05. Pearson’s correlation was used to assess the relationship between anti-fructosylated insulin (IgG) and the two antigens (inhibitors): native and fructosylated insulin. The area under the curve (AUC) and receiver operating characteristic (ROC) curves were used to assess the diagnostic performance of IgGs for T2DM.

### 3.4 Studies on the Cytotoxic Behaviour of Fructosylated Insulin on Human Lymphocytes

#### 3.4.1 Isolation of Peripheral Lymphocytes

After obtaining clearance from the Institutional Ethics Committee (Ref. No: IECJNMC/1036), peripheral venous blood was collected in 0.1 mM EDTA vacuum vials from healthy individuals. Using the density gradient present in Histopaque 1077 (Sigma-Aldrich), lymphocytes were separated. Trypan blue (1%) was used to count cells, and the number of lymphocytes was 1 × 10^6 ± 10^2^.

#### 3.4.2 Quantification of Intracellular ROS

Healthy human lymphocytes from three donors were incubated for 1 hour at 37 °C with 10 µM DCFH-DA, then washed with PBS by centrifugation. After washing, 56 µM native insulin and insulin fructosylated by 5- and 25 mM fructose were mixed with lymphocytes for 20 min. The samples were then diluted 50 times using PBS (pH 7.4). The cells were visualized under a ZEISS Axiocam fluorescence microscope (Germany) at 40X magnification using an excitation wavelength of 485 nm [29]. Experiments were replicated for their reproducibility.

#### 3.4.3 Nucleic Acid Damage

Propidium Iodide (PI) was used to detect the nucleic acid damage in isolated human lymphocytes upon treatment with native or fructosylated insulin samples. Briefly, 75 µM of PI was prepared in 0.1% tri-sodium acetate. The solution was diluted in the ratio 1:3 using PBS. Twenty microliters of this solution was added to the lymphocytes, which had already been treated with different insulin samples for 1 hour at 37 °C. The slides were prepared after washing for 20 min (twice) and visualized under the fluorescence microscope at 40X magnification [30]. Contrast adjustment and image equalization are represented as histograms. Here, the X-axis represents the intensity values (from 0 to 255 for grayscale images) in an 8-bit grayscale image. The Y-axis represents the number of pixels per intensity value after spatial calibration of the image at a pixel aspect ratio of 1, with a known distance of 5 μm. The pixel-to-scale aspect ratio is set at 96 pixels per nanometre.

## 4 RESULTS

### 4.1 Epitope Changes and Characterization of Fructosylated Human Insulin

Native insulin and insulin samples incubated with increasing fructose concentrations showed a concentration-dependent increase in absorbance and a decrease in fluorescence intensity. A maximum increase in absorbance was 55.0%, and a decrease in fluorescence intensity was 53.1%. AGE-specific fluorescence ranged from 21.55% to 33.5%, and the Tht fluorescence ranged from 1.6% to 197.4%. The AGE-specific and ANS fluorescence were not significantly different between insulin fructosylated by 25 and 50 mM fructose. These findings suggest that fructosylation of insulin affects the molecule's overall hydrophobicity, making it more hydrophobic and promoting aggregation. Fructosylation induces oxidative stress, indicated by an increase in carbonyl content by 79%, 123%, 146%, and 176% compared to native insulin. Upon fructosylation with 5-, 10-, 25-, and 50-mM fructose, the absorbance at 420 nm decreased by 19.7%, 29.7%, 35.1%, and 48.6%, respectively, reflecting the loss of free ε-amino groups in lysine residues. A concentration-dependent loss of arginine was also observed (Table **1**). These structural changes led to alterations in the native conformation of insulin. The MRE values obtained at 208 and 222 nm were the average of three replicated CD experiments. The spectra show the deviations in the MRE of the fructosylated insulin samples from the MRE value of the native insulin (Fig. **1**). This data was fed into an online deconvolution software, K2D2. Deconvolution showed 39.37% alpha helix, 11.52% beta sheet, and 49.11% unordered structures in native insulin. Furthermore, fructosylation reduced alpha-helix content and increased beta- and unordered-structure content (Table **1**), thereby promoting insulin aggregation and perturbing its native structure and conformation. These changes represent a shift in insulin's structural and conformational profile, potentially transforming it into an antigenic protein under hyperglycemic conditions. Hyperglycemia stimulates endogenous fructose production, making insulin more susceptible to fructosylation.

### 4.2 Direct Binding ELISA and Quantification of Antibody Titer

Healthy sera exhibited the least binding with native and fructosylated insulin. Sera from T2DM sera also showed less interaction with the native insulin. However, T2DM sera exhibited stronger binding when fructosylated insulin was used as an antigen. Antibodies present in T2DM recognized fructosylated insulin significantly more than native insulin at *p*=0.001 (Fig. **2**). T2DM sera did not show any quantifiable titer for native insulin, whereas they exhibited an antibody titer of > 1:1600 against fructosylated insulin (Fig. (**3**). The data obtained by direct binding ELISA were either normally distributed or symmetrically presented (Table **2**). To understand the binding specificity of the serum samples, inhibition ELISAs were performed using isolated IgGs from T2DM serum samples (NHS samples did not show a significant response in direct ELISA, hence inhibition studies were done on T2DM IgGs only).

### 4.3 Descriptive Statistics of the Data Obtained from Direct Binding ELISA

The descriptive statistical analysis of data obtained from direct binding ELISA of normal and T2DM sera against native and fructosylated insulin showed that the 75th percentile of the data recorded binding absorbance values: 0.223, 0.163, 0.1828, and 0.8435. The first two values correspond to the binding of NHS to native and fructosylated insulin, and the other two to T2DM sera binding to native and fructosylated insulin. The data related to the binding of fructosylated insulin with NHS and T2DM sera were negatively skewed with small Kurtosis. The remaining parameters are shown in Table **3**.

### 4.4 Isolation of IgGs from T2DM Serum Using Protein A Agarose Affinity Chromatography

IgGs were isolated from the sera of T2DM patients, showing appreciable interaction during direct binding ELISA (n = 20). Fractions were collected and checked for maximum absorption at 280 nm. The purified IgGs were pooled and subjected to a 7.5% SDS-polyacrylamide gel (in duplicates) under non-reducing and reducing conditions (Fig. **4**).

### 4.5 Inhibition ELISA

An inhibition ELISA was used to assess the binding specificity of the isolated antibodies (IgG) for native and fructosylated insulin. Percent inhibition with native insulin as the antigen at 20 μg/ml ranged from 23.8% to 40.5%, with a mean ± SD of 31.4 ± 5.1%. However, when fructosylated insulin was used at the same concentration, maximum percent inhibition ranged from 47.1% to 63.1% with a mean ± SD of 55.7 ± 3.7% (Fig. **5** and Table **4**). The mean percent inhibition and Pearson’s correlation with linear regression are depicted in Fig. (**5A-E**), respectively.

### 4.6 Statistical Analysis of the Sensitivity, Specificity, and Predictive Value for the Presence of Anti-Fructosylated IgGs

The statistical probability of screening for anti-fructosylated insulin antibodies as a diagnostic measure was evaluated using receiver operating characteristic (ROC) analysis and the Area Under the Curve (AUC) Fig. (**6**). The affinity of serum IgG obtained from T2DM showed a variable degree of absorbance (0.2-0.892), with a mean ± SD of 0.695 ± 0.223. The mean % inhibition in the competitive ELISA was 55.7±3.7 for fructosylated insulin, compared with 31.4±5.1 for native insulin as the antigen. The binding sensitivity and specificity of IgG isolated from sera of T2DM patients with human insulin fructosylated with 50 mM fructose, and the plot of sensitivity vs 1-specificity showed AUC=1.000 at *p* < 0.01, at 95% CI. The likelihood ratio at 100% sensitivity and 96.67% specificity was 30.00. Therefore, it provides strong evidence that the presence of anti-fructosylated insulin antibodies in human serum has high predictive value for T2DM. Further investigation using a larger sample size may help to establish the findings.

### 4.7 Analysis of the Cytokines Involved in the Subclinical Autoimmune Responses

Cytokines are small, biologically highly active proteins that regulate the growth, function, and differentiation of cells and help steer the immune response and inflammation. In the case of the NHS, the concentrations of IL-6 were 3.62±1.35 pg/ml, IL-10 was 25.62±4.31 pg/ml, and IL-17 was 4.25±0.99 pg/ml, and IL-18 was 27.20±4.28 pg/ml. Whereas in the case of T2DM, the concentrations of the mentioned cytokines were IL-6, 9.19±1.84 pg/ml; IL-10, 12.30±3.39 pg/ml; and IL-18, 54.15±6.68 pg/ml. The *p*-value between the NHS and T2DM in all the cases was < 0.000. IL-17 was found to be 6.77±0.782. The *p*-value between the NHS and T2DM in this case was < 0.05 Fig. (**7**).

### 4.8 Analysis of the Cytotoxic Nature of Fructosylated Human Insulin

#### 4.8.1 Intracellular ROS Detection and Nucleic Acid Damage

Fluorescence microscopy at 40X magnification revealed that the native insulin did not alter the oxidative environment of the lymphocytes. Thus, the micrographs did not exhibit any DCFH-DA-related fluorescence (Fig. **8A**). However, when lymphocytes were preincubated with fructosylated insulin (5 mM fructose) and subjected to a DCFH-DA assay, the cells showed little green fluorescence, as is typical for ROS detection with DCFH-DA (Fig. **8B**). Lymphocytes treated with fructosylated insulin (25 mM) exhibited a strong green fluorescence with increased intensity specific to DCFH-DA (Fig. **8C**).

Nucleic acid damage in isolated peripheral lymphocytes was assessed with native and modified insulin treatment. Propidium iodide was used in this study. Upon PI staining, lymphocytes preincubated with native insulin showed no fluorescence (Fig. **8D**). However, when lymphocytes were treated with fructosylated insulin (5 and 25 mM fructose), red-colored fluorescence was detected in a concentration-dependent manner (Fig. **8E** and **F**). This indicated membrane disruption and irreversible DNA damage, indicating a progression towards apoptotic cell death.

## 5 DISCUSSION

Type 2 diabetes mellitus (T2DM) remains a serious metabolic disturbance condition with an interlinked complicated pathologies resulting from insufficient insulin function, release, and/or the disrupted β-pancreatic cells [31]. Among the closely associated risk factors, obesity serves as the profound cause of diabetes. It is usually driven by a sedentary lifestyle accompanied by the absence of physical activity and sustained dietary caloric surplus. Overconsumption of hypercaloric sugars is one of the reasons for caloric surplus and is often linked to obesity-induced T2DM [32]. Regular consumption of foods containing additional sweeteners, such as HFCS, along with a sustained sedentary lifestyle inevitably accelerates the metabolic derangements that create a pro-diabetic scenario [33]. Overconsumption of fructose, a key ingredient in the sweetened beverage industry, seriously impairs metabolism and increases the risk of T2DM. Apart from the impaired glucose tolerance, fructosylation of proteins is also unavoidable in a high-fructose environment. Protein products of fructosylation or glycation result in the accumulation of AGEs as their products. AGEs formed cause complications in small vessels, such as blood capillaries, and large blood vessels, such as arteries and veins, leading to vascular pathologies [14]. Since insulin is the key peptide hormone in glucose homeostasis, a compromise in its structural, biochemical, and conformational properties would compromise its functionality. In diabetes, uncontrolled glucose and fructose levels render proteins more prone to glycation or fructosylation. The cellular uptake of fructose by islets of Langerhans may create a potential opportunity for fructosylation of insulin during its early stages of synthesis, thus increasing the likelihood of fructosylated insulin secretion into the blood. Upon release, a physicochemically compromised insulin molecule can further contribute to the existing insulin resistance. This modified insulin may also self-present as an antigen, eliciting an immune response that includes the production of autoantibodies. Thus, this study studied the presence of anti-fructosylated IgGs in the sera of diabetic patients by using fructosylated insulin as an antigen. Additionally, the toxic effects of fructosylated insulin on lymphocytes were probed.

Physicochemical studies were conducted on insulin fructosylated with 5, 10, 25, and 50 mM fructose. Native insulin was used as a control. Results from UV spectroscopy indicated that incubation with fructose resulted in a concentration-dependent, progressive increase in hyperchromicity at 276 nm, suggesting possible unfolding of the insulin structure. Fructosylation exposes aromatic amino acid residues that are buried within the globular structure of insulin, thereby favoring biochemical changes that lead to protein unfolding or the formation of aberrant secondary structures [34]. A spectrofluorometer measured tyrosine fluorescence. To study the intrinsic fluorescence properties of native and insulin incubated with fructose, samples were excited at 275 nm, and maximum emission was recorded at 305 nm. The fluorescence intensity of native insulin showed quenching behavior in the presence of fructose, again in a concentration-dependent manner.

Fructosylation of proteins is a slow process in which fructose reacts with proteins, generating AGEs [35]. Since insulin was incubated with increasing fructose concentration, the formation of AGEs was expected. Native insulin did not show any AGE-specific fluorescence. However, AGE-specific fluorescence was detected in all samples incubated with fructose at all the mentioned concentrations. These results suggest that prolonged exposure of insulin to fructose can contribute to the overall production of AGEs in the body and to the production of physiologically dysfunctional insulin. The protein carbonyl group was used as a marker of AGEs-related oxidative stress [36]. An increase in the carbonyl content of insulin molecules was observed upon fructosylation. The changes in the carbonyl levels indicated the oxidation of insulin. Furthermore, the chemical analysis was done on fructosylated insulin to estimate free lysine and arginine residues. Since these two amino acids are most susceptible to biochemical modifications, modifications at these sites would further aggravate insulin's structural alterations. Fructosylation significantly reduced the free lysine and arginine content in a concentration-dependent manner. Lysine and arginine residues are located on the surface of protein molecules and provide structural stability by participating in ionic interactions, hydrogen bonding, and interactions with water molecules [37, 38]. Thus, modification of the ε-amino groups suggests compromised structure and functionality. This is expected to interfere with insulin secretion, insulin signaling, and self- and non-self-discrimination by the immune system.

Deviations in the chemistry of a protein from its native state are reported to be responsible for its functional loss and, hence, associated with serious medical conditions [39]. The increase in UV absorbance, quenching of intrinsic fluorescence, increased carbonyl content, reduction in free lysine and arginine residues, and increase in fructosamine content indicated the possibility of gross structural perturbations in the insulin molecule upon fructosylation, which could lead to an aggregating tendency and new epitope presentations. ANS is a fluorophore probe highly sensitive to a protein's exposed hydrophobic sites [40]. Compared to native insulin, there was an abnormal rise in the ANS-specific fluorescence intensities obtained for fructosylated insulin samples. This rise was attributed to the fructose-induced exposure of the hydrophobic sites in the insulin structure. The hydrophobic regions of the protein are clustered within the protein molecule to provide stability by reducing its surface area and minimizing interactions with water molecules. Exposing hydrophobic sites increases the likelihood of protein aggregation. Thus, the exposed hydrophobic sites of insulin due to fructosylation might have left insulin susceptible to aggregation.

The rise in ThT-specific fluorescence was observed when fructosylation was induced at 50 mM of fructose. This indicated the presence of amyloid-like structures. This implied that chronic high-fructose exposure not only affected the biochemical, structural, and conformational properties but also increased insulin's tendency to aggregate in an aqueous environment and to progress toward fibril formation, leading to amyloid-like accumulations.

Further, to evaluate the alpha-helical content in insulin molecules, CD spectroscopy performed on native and fructosylated insulin samples revealed that at all fructose concentrations, fructosylation induced a progressive loss (compared to the native insulin) in the MRE at 208 and 222 nm in the near-UV range. These results corresponded to a concentration-dependent loss in alpha-helicity of insulin upon fructosylation. The detailed analysis of the CD data was performed using K2D2 (online software for CD deconvolution). It was found that a concurrent increase in β-turns and unordered structures accompanied the loss in total alpha-helical content. Thus, fructosylation induced secondary structural alterations in insulin that could lead to functional loss. In proteins, secondary structural modifications are usually followed by the formation of aggregates organized in β-sheet complexes [41]. Insulin in a polymerized state loses its biological activity [42]. Once the presence of aggregates was ascertained, further morphological studies were conducted, and hydrodynamic radii were determined by performing DLS. The increase in R_h_ was dependent on fructose concentration. Compared to native insulin, there was a significant increase in R_h_ for insulin fructosylated at 10 and 50 mM fructose. From DLS studies, it was concluded that the aggregates formed were significantly larger than those of native insulin. This was in agreement with the ThT findings. These results show that fructosylation alters insulin chemistry, leading to structural, conformational, and morphological deformities.

Glycated/fructosylated insulin remains in circulation in patients suffering from T2DM [43, 44]. Since fructose levels are also elevated in T2DM, the probability of insulin undergoing fructosylation is therefore always high in such patients. Hence, insulin could be secreted as a fructosylated product or may later undergo fructosylation. Fructosylated insulin, like other proteins, presents with altered surface properties [20, 21, 45-48]. Such protein fructosylation products may be recognized as non-self by the immune system and are expected to induce specific immune responses (Fig. **9**). This phenomenon is expected to vary from patient to patient. Since most of the patients of T2DM are either accidentally diagnosed or remain undiagnosed [49], screening diabetic patients for the presence of anti-fructosylated insulin antibodies could prove beneficial in terms of a personalized approach or prognostic relevance. In this regard, sera from patients diagnosed with T2DM conditions (according to ADA) were screened for the presence of circulating anti-fructosylated insulin IgGs.

Data from a direct binding ELISA showed that sera from diabetic patients exhibited a significantly stronger binding affinity for anti-fructosylated insulin than for native insulin. The presence of neo-epitopes was anticipated from these findings, which confer immunogenicity. The low-binding values of the healthy sera against fructosylated insulin reassure the formation of anti-fructosylated insulin IgGs in diabetic patients at any time. Analysis of the descriptive statistics showed that 75 percent of T2DM sera contained anti-fructosylated insulin IgGs with significantly higher binding affinity. Upon confirmation of the presence of anti-fructosylated antibodies in diabetic sera, IgGs were isolated from the sera of diabetic and control samples. An appreciable UV absorbance peak of the eluted IgGs was observed at 280 nm, which corresponded to the absorption of UV light by aromatic groups of the amino acids. Analysis from the SDS-PAGE under reducing and non-reducing conditions was also performed. In non-reducing conditions, a single dense, heavier band was observed corresponding to the intact IgGs. However, under reducing conditions, single light and heavy bands (in duplicates) were observed. The direct binding ELISA showed the titer of the antibodies to be 1:1600 in the case samples. Analysis of the inhibition ELISA showed a strong binding affinity (55.7±3.7%) towards fructosylated insulin and 31.4±5.1% for native insulin. These results agree with the previous findings observed in direct binding ELISA and confirm the specificity and cross-reactivity of T2DM-IgG. Pearson’s correlation and linear regression plots with respect to inhibitor concentrations (μg/ml) showed a higher R^2^ value for fructosylated insulin. This highlights the better linear relationship between the two variables. If the data are normally distributed, the R^2^ value is 1, and the relationship is linear. Analysis of the ROC curve showed an AUC of 1, indicating a strong model that differentiates between diseased and non-diseased individuals. Furthermore, at 95% confidence, the isolated IgGs were found to be 96.67% specific and 100% sensitive towards fructosylated insulin. Finally, a likelihood ratio of 30 was also recorded, defined as the ratio of the probability of a positive result in a diseased condition to the probability of a false positive (*i.e.*, the absence of a disease). T2DM serum samples showed elevated IL-6, IL-17, and IL-18 values and reduced IL-10 values. The findings are consistent with ELISA results, indicating a proinflammatory status.

Amorphous aggregates and amyloid-like fibrils are cytotoxic to the cells and are a source of oxygen-free radicals [43, 50]. To understand the effect of the aggregates of fructosylated insulin and their pro-oxidative nature, they were exposed to isolated human lymphocytes, the primary immune cells. The generation of free oxygen radicals was visualized using a DCFH-DA assay. Lymphocytes treated with native insulin appeared normal, as no DCFH-DA-specific (green) fluorescence was observed. However, a strong green fluorescence was observed in lymphocytes incubated with fructosylated insulin samples, confirming increased ROS activity. From this, it was asserted that fructosylation induces cytotoxic insulin aggregates that, upon contact with lymphocytes, may initiate a concentration-dependent cascade of ROS generation. The accumulation of ROS and associated oxidative stress is a well-established precursor to nucleic acid damage. Therefore, a PI assay was performed to identify nucleic acid damage. The uptake of PI shows that lymphocytes treated with fructosylated insulin exhibited red fluorescence. Fructosylated insulin (at 50 mM fructose) appeared to induce apoptosis in exposed lymphocytes.

## CONCLUSION

Autoimmunity, which defines self-reactivity, is not limited to immune-mediated tissue damage; rather, it can range from physiological homeostasis to the presence of circulating autoantibodies and deleterious, frank autoimmune responses. The current classification of T1DM, T2DM, and latent autoimmune diabetes of the young (LADA) presents challenges for the diagnosis and treatment of diabetic patients. LADA is often diagnosed in adults and initially managed like Type 2 diabetes. The findings of this study corroborate the TJ Wilkin accelerator hypothesis of 2001. Accumulating evidence suggests that overnutrition and high-fructose diets alter insulin signaling. The present study also provides evidence that fructose can modify insulin's native structure and conformation, presenting neo-epitopes on its surface, which may alter immune cell responses. Although the presence of anti-fructosylated insulin antibodies may not support a classic autoimmune phenomenon, they are consistent with some features of the inflammatory trajectory observed in all stages of T2DM and are likely attributable to low-grade, subclinical, autoimmune activity. The role of autoimmune phenomena in T2DM could be masked by the disease's multifactorial nature and heterogeneity. This study may open pathways to a better understanding of autoimmunity in T2DM. Identifying the immunogenic basis of the disease can help distinguish patient subgroups for mechanism-based treatments. It may also aid in developing new strategies to prevent or manage T2DM development, progression, and related complications. The sample size was limited to 80 participants, which could be expanded to improve the study's statistical power. Including cases of T1DM would also help differentiate and compare the presence of anti-fructosylated insulin antibodies in T2DM and T1DM. The goal of this research was to identify potential antigens involved in low- grade, chronic inflammation in T2DM, which occurs without major signs of acute inflammatory response. The findings are exploratory and may reflect residual confounding, including the duration of diabetes and associated chronic complications. Prospects of the study may include mass spectrometry (MS) or MS/MS confirmation of modification sites on fructosylated insulin and the identification of the relationship between the effects of antidiabetic drugs and fructosylation, in light of indices of insulin sensitivity/insulin resistance. An extension of this work may also focus on identifying the association between inflammatory mediators and the fructosylation process.

## Figures and Tables

**Fig. (1):**
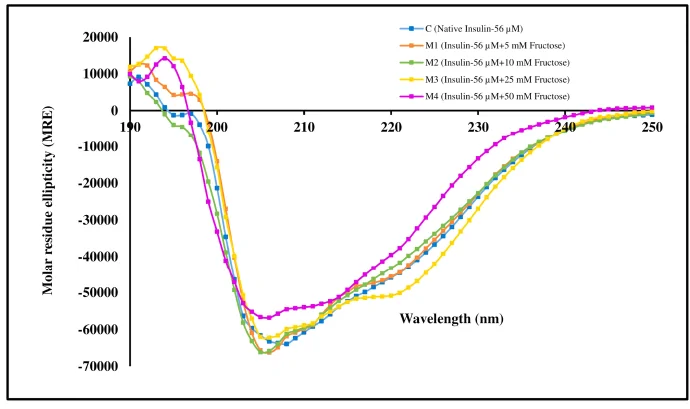
CD spectra of native insulin (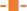) and insulin incubated with 5 mM (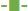), 10 mM (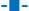), 25 mM (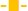), and 50 mM (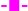) fructosylated 37ºC for 10 days.

**Fig. (2):**
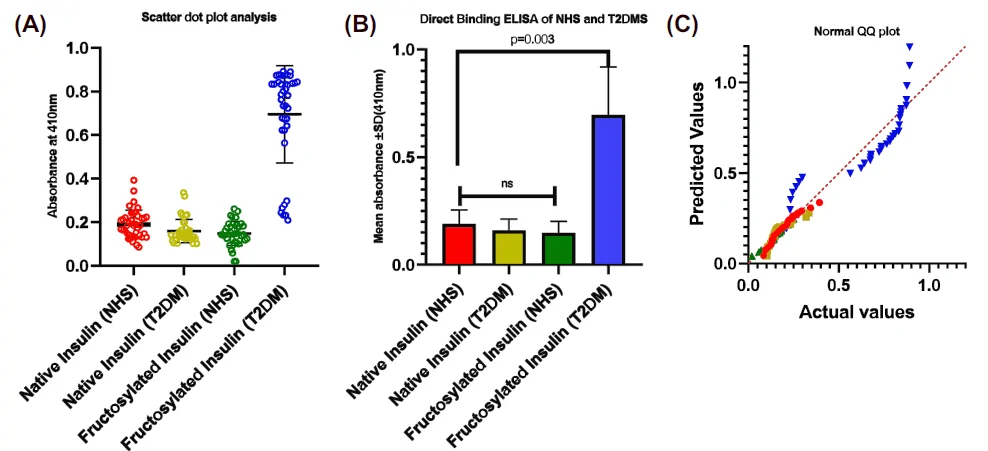
Scatter dot plot analysis (**A**), bar graph analysis (**B**), and normal QQ plot (**C**) of direct binding ELISA for native insulin with normal human sera (NHS) (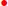), native insulin with T2DM sera (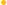), fructosylated insulin with NHS (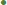), and fructosylated insulin with T2DM sera (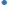).

**Fig. (3):**
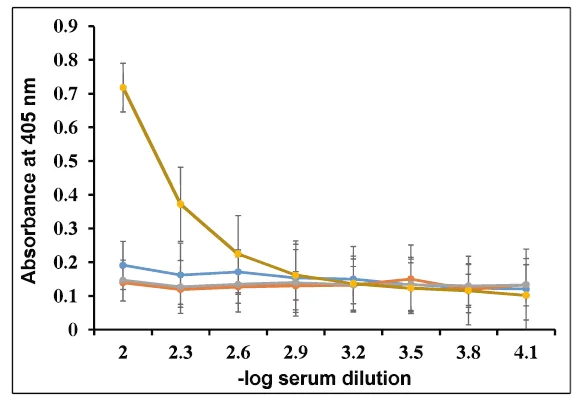
Direct binding ELISA to check the titer of IgG in sera from healthy humans against native (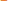) and fructosylated insulin (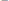) and T2DM patients’ sera against native (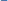) and fructosylated insulin (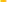). The microtiter plates were coated with the respective antigens (10 μg/ml).

**Fig. (4):**
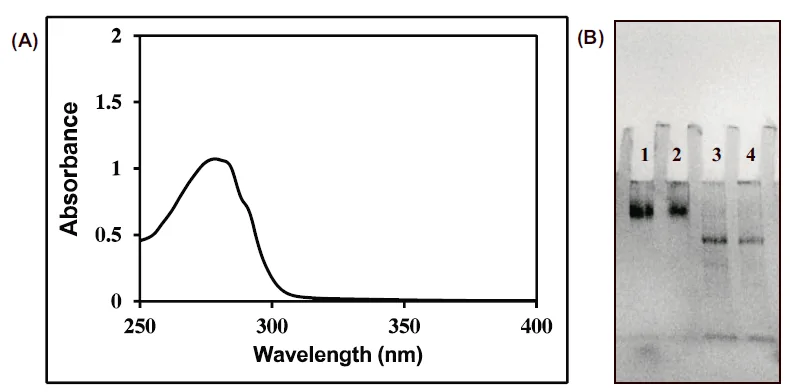
Elution profile of affinity-purified human IgG (**A**). Purified IgG (in duplicate) on a 7.5% SDS-PAGE in non-reducing (lanes 1 and 2) and reducing conditions (lanes 3 and 4) (**B**).

**Fig. (5):**
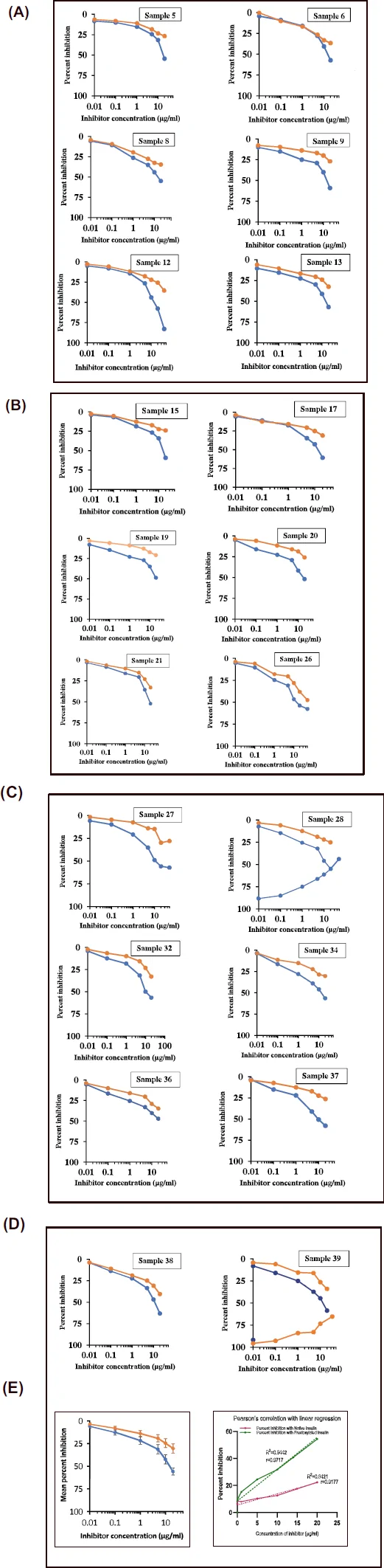
Inhibition ELISAs of best binding T2DM sera against native (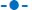) and fructosylated insulin (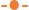). Here, native and fructosylated insulin (0-20 µg/mL) was used as inhibitors. Microtiter plates were coated with 10 µg/ml of respective antigens. This figure (**A** to **D**) represents samples Nos. 5, 6, 8, 9, 12, 13, 15, 17, 19, 20, 21, 26, 27, 28, 32, 34, 36, 37, 38, and 39 from the direct binding ELISA software data lists. Mean percent inhibition of serum autoantibody binding in T2DM patients against native insulin (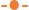) and fructosylated insulin (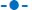), and Pearson’s correlation with linear regression plot with respective R^2^ and r values for percent inhibition with native (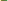) and fructosylated insulin (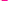) (**E**).

**Fig. (6):**
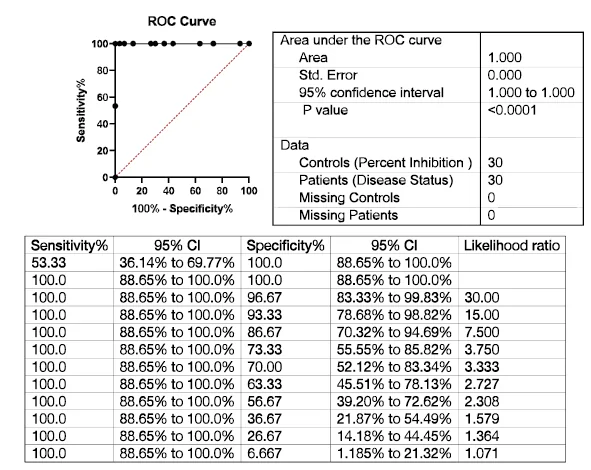
ROC and AUC analysis for the sensitivity and specificity of IgG isolated from T2DM against fructosylated insulin (50 mM fructose).

**Fig. (7):**
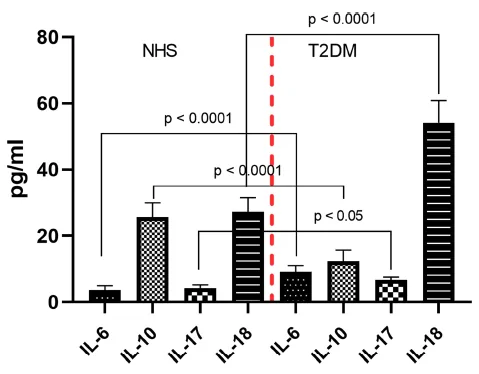
Mean ± SD of cytokine concentration in serum samples from NHS and T2DM subjects. The red dotted line separates the two groups. NHS stands for Normal Human Serum, and T2DM for type 2 diabetes mellitus.

**Fig. (8):**
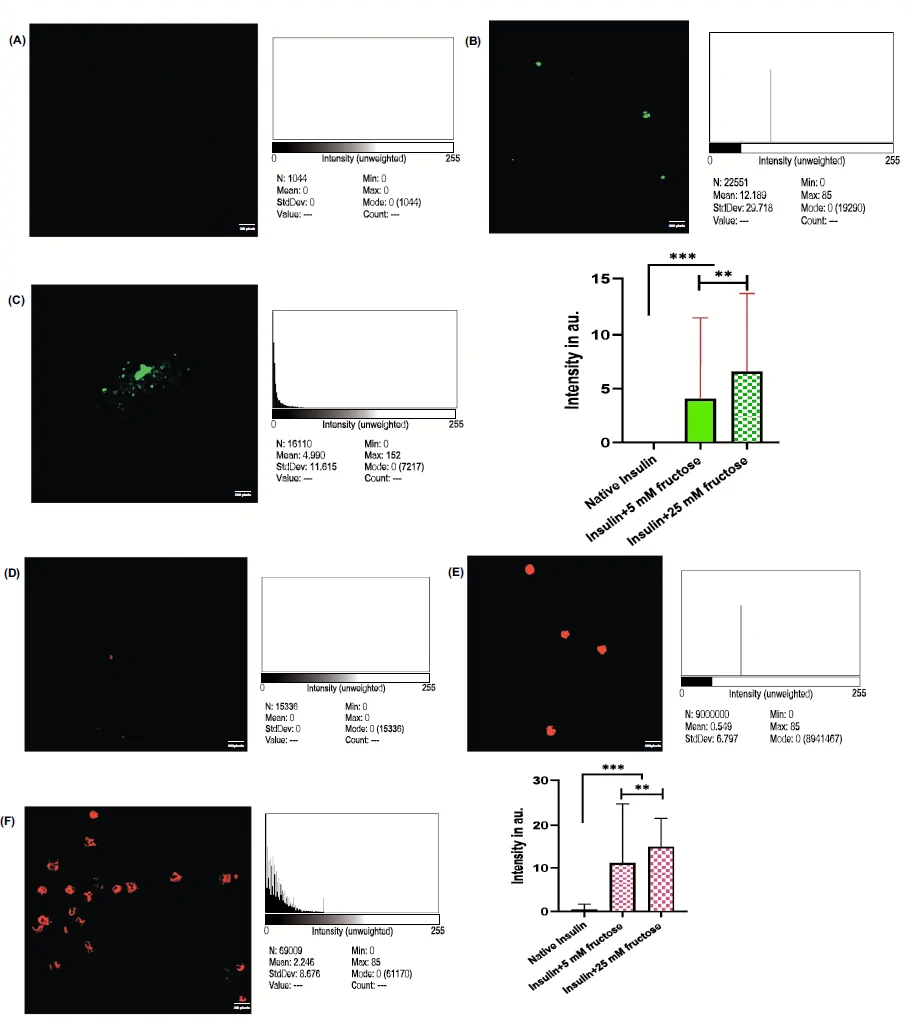
Fluorescence micrographs of human lymphocytes treated with native insulin (**A**), insulin fructosylated with 5 mM fructose (**B**), and insulin fructosylated with 25 mM fructose (**C**). 10 µmol of DCFH-DA was added to the samples (A-C), and micrographs were captured at 40X magnification after excitation at 485 nm. Fluorescence micrographs of human lymphocytes treated with native insulin (**D**), insulin fructosylated with 5 mM fructose (**E**), and insulin fructosylated with 25 mM fructose (**F**). Twenty µL of 75 µM propidium iodide was added to the samples (**D** to **F**), and the samples were incubated at 37 ºC. The micrographs were captured at 40X magnification after exciting the samples at 535 nm. ** indicates *p*-value ≤ 0.05 and *** indicates *p*-value ≤ 0.0001.

**Fig. (9):**
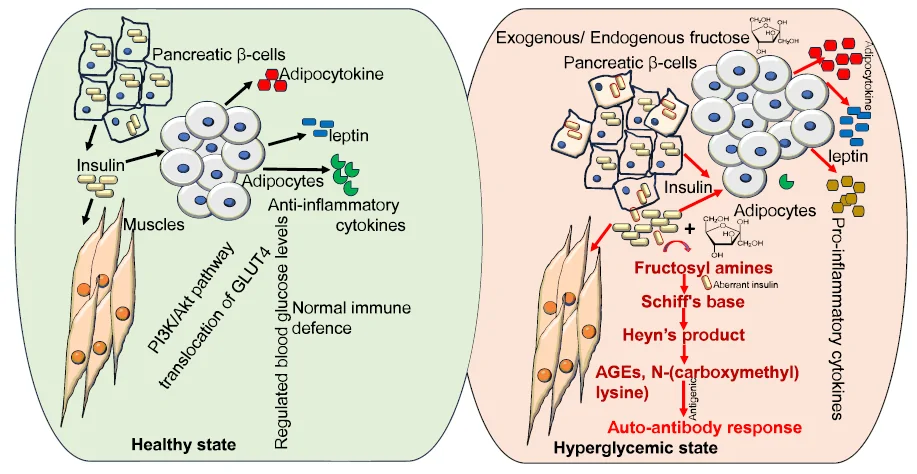
The subclinical inflammatory elements of type 2 diabetes caused by high fructose levels are depicted here. The figure illustrates the possible rise of cryptic insulin acting as a self-antigen. This abnormal insulin molecule could trigger increased insulin secretion, reduced responsiveness, and worsening dysregulation of glucose levels.

**Table 1:** Structural, biophysical, and conformational assessment of human insulin treated with fructose.

**Parameters**	**Native** **Insulin**	**Insulin + 5 mM** **Fructose**	**Insulin + 10 mM** **Fructose**	**Insulin + 25 mM** **Fructose**	**Insulin + 50 mM** **Fructose**
**Change in UV-vis absorbance at 276 nm**	-	0.8%	10.0%	23.3%	55.0%
**Percent change in intrinsic fluorescence**	-	21.2%	41.8%	43.3%	53.1%
**AGE-specific fluorescence at 440 nm**	-	21.5%	22.8%	30.4%	33.5%
**Percent increase in ThT fluorescence at 450 nm**	-	1.6%	17.1%	26.9%	197.4%
**Percent increase in ANS fluorescence at 480 nm**	-	300%	312%	340%	360%,
**Percent loss of ε-amino group of the lysine at 420 nm**	-	19.7%	29.7%	35.1%	48.6%,
**Percent loss of free arginine residues at 395 nm**	-	14.2%	20.1%	46.4%	58.8%
**Protein carbonyl (nmole/mg insulin)**	1.30±0.029	2.33± 0.047	2.98±0.034	3.26±0.049	3.69±0.81
**DLS (hydrodynamic radii) nm**	2.48±0.38	84.9±4.08	98.6±3.72	607±12.02	637±62.44
**Polydispersity index (PDI)**	0.440	0.673	0.753	0.802	0.903
**Units of MRE observed at 208 and 222 nm**	−63945.3 and − 41265.4	−61879.5 and −42375.4	−61231.1 and −39783.4	−59903.4 and −48623.9	−54524.7 and −35229.7
α-helix*	39.37%	29.6%	19.7%	15.43%	10.45%
β-Strand*	11.52%	17.5%	24.6%	26.43%	29.09%
Unordered structures*	49.11%	52.9%	55.7%	58.14%	60.46%

AGE: Advanced glycation end-products, ThT: Thioflavin T, ANS: 8-anilino-1-naphthalenesulfonic acid, DLS: Dynamic Light Scattering, MRE: Molar residue ellipticities.

*Changes in the helicity were calculated using K2D2, an online software for estimating protein secondary structure from the circular dichroism spectra.

**Table 2:** The table presents the results of the Kolmogorov-Smirnov (KS) test for normality in the direct binding ELISA.

	**Native Insulin (NHS)**	**Native Insulin (T2DM)**	**Fructosylated Insulin (NHS)**	**Fructosylated Insulin (T2DM)**
**KS distance**	0.08118	0.2555	0.07307	0.20009
***p* value**	>0.1000	<0.0001	>0.1000	0.0003
**Passed normality test (alpha=0.05)?**	Yes	No	Yes	No
***p*-value summary**	ns	****	ns	***
**Number of values**	40	40	40	40

**Table 3:** Descriptive analysis of data obtained from direct binding ELISA of normal and T2DM sera against native and fructosylated insulin.

**Number of Values (n=40)**	**Native Insulin (NHS)**	**Native Insulin (T2DM)**	**Fructosylated Insulin (NHS)**	**Fructosylated Insulin (T2DM)**
**Absorbance range at 410 nm**	(0.086−0.393) 0.307	(0.102−0.335) 0.233	(0.020−0.261) 0.241	(0.210−0.892) 0.682
**Mean ± Standard deviation of absorbance**	0.1899 ± 0.06526	0.1596 ± 0.05303	0.1477 ± 0.05458	0.6954 ± 0.2233
**Standard error of the mean**	0.01032	0.008385	0.008629	0.03531
**Median**	0.1837	0.1448	0.1451	0.7930
**Skewness/Kurtosis**	1.017/1.453	1.867/3.579	-0.2353/0.365	-1.298/0.266

NHS represents normal human serum, and T2DM represents type 2 diabetes mellitus serum. Native and fructosylated insulin are two antigens used in ELISA.

**Table 4:** Maximum percent inhibition of serum autoantibodies from T2DM sera at 20 µg/ml of native and fructosylated insulin used as inhibitors.

**Serum Sample No.s**	**Maximum % Inhibition Obtained with Native Insulin** **(20 µg/ml)**	**Maximum % Inhibition Obtained with Fructosylated Insulin** **(20 µg/ml)**
5	26.6	54.3
6	36.6	57.2
8	34.0	54.7
9	27.5	59.0
12	25.5	57.5
13	32.3	56.9
15	23.8	59.2
17	30.9	60.5
19	28.6	48.6
20	25.7	51.7
21	33.1	52.3
26	38.0	53.7
27	29.7	55.7
28	24.1	54.9
32	32.8	56.5
34	38.3	56.4
36	34.7	47.1
37	26.2	57.8
38	40.5	63.1
39	34.05	58.5
**Mean ± SD**	**31.4 ± 5.1**	**55.7 ± 3.7**

## Data Availability

All data generated or analyzed during this study are included in this published article.

## LIST OF ABBREVIATIONS

T2DM: Type 2 Diabetes Mellitus
AGEs: Advanced Glycation End-products
IDF: International Diabetes Federation
YOD: Young-Onset Diabetes
BMI: Body Mass Index
HFCS: High-Fructose Corn Syrup
